# AACN COMPETENCY-BASED ESSENTIAL REVISIT: EVIDENCE-BASED VALIDATION VIA RANDOMIZED CONTROL TRIAL

**DOI:** 10.1093/geroni/igae098.0172

**Published:** 2024-12-31

**Authors:** Hsueh-Fen Kao, Shirin Kiani

**Affiliations:** The University of Texas El Paso, El Paso, Texas, United States; Green Mountain Hospice, El Paso, Texas, United States

## Abstract

In April 2021, American Association of Colleges of Nursing (AACN) approved The Essential for Professional Nursing Education, which calls for preparing future nurses using a competency-based approach in designing curricula for schools of nursing. Taiwan’s nursing education system follows the American Model. This study, conducted in fall 2022 to spring 2023, intended to validate the competency-based approach through entrustable professional activities (EPAs) in the yet widely used nursing education arena. EPAs are practical approaches in assessing competence in real-world settings. A randomized control trial was used in Registered Nurse to Bachelor of Science in Nursing (RN-BSN) students at a college in southern Taiwan. ‘Wound care for older adults with diabetes’ was chosen to be the EPA intervention. After random assignment, students in the experimental group (n=31) received extra EPA simulation laboratory practice with feedback, and those in the control group (n=34) received usual practice and evaluation without simulation laboratory practice and feedback. In the end of semester, data in Competency Inventory of Nursing Students, Learning Satisfaction, Objective Structured Clinical Examinations were collected using analysis of variance (ANOVA) and Student’s t test for analysis. The experimental group demonstrated significantly higher scores in competency and clinical examinations than the control group. However, both groups experienced an increase in learning satisfaction without reaching a significant difference, probably due to receiving more attention. Results of the study indicate that evidence-based pedagogy, e.g., EPAs, can serve as effective strategies in designing competency-based curricula in nursing education to meet the new AACN standards with compelling evidence.

